# The Oncogenic Potential of Human Papillomavirus in Relation to Multiple Types of Cancer in Saudi Arabia: A Systematic Review

**DOI:** 10.7759/cureus.57851

**Published:** 2024-04-08

**Authors:** Mashael S Alfaifi

**Affiliations:** 1 Department of Epidemiology, Faculty of Public Health and Health Informatics, Umm Al-Qura University, Mecca, SAU

**Keywords:** head and neck cancer, ovarian cancer, colon cancer, cervical cancer, human papillomavirus (hpv)

## Abstract

The oncogenic potential of human papillomavirus (HPV) has been widely acknowledged in relation to multiple types of cancer. The objective of this investigation was to conduct a comprehensive assessment of the available evidence pertaining to the correlation between HPV and various types of cancer, such as cervical, colon, ovarian, and head and neck cancers, in the Kingdom of Saudi Arabia (KSA).

The Preferred Reporting Items for Systematic Reviews and Meta-Analyses (PRISMA) guidelines were complied with to conduct a systematic literature search aimed at identifying studies that explore the correlation between HPV and the specified cancers. Databases such as Web of Science, Embase, PubMed, and the Cochrane Library were queried up until May of 2023. Relevant literature was obtained, information was extracted, and the methodological rigor was evaluated.

The high-risk HPV, namely HPV-16 and HPV-18, were detected as the most prevalent variants in KSA. A significant proportion of cervical cancer cases in the region were found to be associated HPV infection. The molecular tests have furnished evidence that establishes a connection between HPV infection and colonic polyps as well as colorectal cancer. This finding suggests that HPV may have a plausible role in the etiology of these medical conditions. The results of genotyping and integration analyses suggest a probable correlation between HPV and the development of ovarian cancer. Additionally, the prevalence of head and neck squamous cell cancer related to HPV was notably reduced in this particular geographical area.

This study presents persuasive findings that establish a connection between HPV and cervical cancer and proposes plausible correlations with squamous cell carcinomas in the colon, ovaries, and head and neck. The aforementioned results emphasize the necessity for additional inquiry into the function of HPV in the onset and advancement of said malignancies. Further investigations are necessary to augment our comprehension of the role of HPV in these neoplasms.

## Introduction and background

Human papillomaviruses (HPVs) are a class of double-stranded, non-enveloped viruses that are categorized within the *Papillomaviridae* family. The virus is around 52-55 nm in diameter. Capsids are protein shells that surround viral DNA [1]. L1 and L2 are two structural proteins that make up the HPV capsid. The bulk of the capsid is formed by L1, which is responsible for the capsid's structural integrity; L2, which is located inside the capsid, aids in viral entrance into host cells [2]. The viral DNA codes for early (E) and late (L) genes that are essential for the replication of viruses and expression of genes [3]. More than 200 distinct varieties of HPV have been identified and are categorized by DNA sequence and disease interaction. The categorization of these strains into low-risk and high-risk is based on their potential to induce cancer. The development of genital warts is primarily attributed to low-risk HPV strains, specifically HPV-6 and HPV-11. Conversely, it has been established that high-risk strains of HPV, specifically types 16 and 18, are causally linked to the development of cervical, pharyngeal, and other malignancies [4].

Epithelial basal cells are particularly susceptible to infection by HPV, especially in the mouth and genitalia. The virus infects host cells through epithelial damage such as microabrasion. After entering the cell, the virus multiplies and generates viral proteins by releasing its DNA, which is then taken to the cell's nucleus [5,6]. Two distinct phases distinguish the human HPV life cycle: the active phase and the dormant phase. New virus particles are produced by viral replication during the productive phase. The subsequent release of these infectious particles facilitates disease transmission. The DNA of the virus may stay dormant in the host cell during the latent phase, when neither symptoms nor new virus particles are produced. However, the virus might begin its productive phase again under certain circumstances [7,8].

The most commonly observed histological type of cancer linked to HPV is squamous cell carcinoma, which is attributed to the virus's preference for epithelial cells. Cancers of the cervix, vulvar, oropharynx, and anus have all been linked to HPV with varying degrees of certainty [9,10]. Multiple meta-analyses have consistently found a correlation between HPV infection and a reduced propensity for anogenital and head and neck cancers (HNCs) to develop [11,12]. Although the precise mechanism by which the presence of HPV improves prognosis in these tumors remains unclear, it has been discovered that HPV-negative primary cancers have a higher likelihood of metastatic spread and more aggressive p53 mutations, leading to more serious disruption of normal growth regulation and a poorer prognosis [13]. The association between HPV and urological malignancies has been extensively researched for almost 30 years due to the close proximity of anogenital carcinomas. So far, HPV has only been associated with penile cancer. The etiology of prostate, kidney, bladder, and testicular cancers perhaps involves HPV; however, this remains a topic of ongoing debate.

The exponential growth of cells induced by a high-risk HPV infection can result in the development of pre-cancerous changes or neoplastic growths in the event of immune system inadequacy [14]. Apart from the high-risk HPV types, various factors contribute to an elevated probability of the advancement from precancerous cervical cells to invasive cancer. Several factors have been identified as potential contributors to the development of cervical cancer, including extended use of oral contraceptives, multiple occurrences of pregnancy, smoking, weakened immune function, co-occurrence of other sexually transmitted infections (STIs), and increased age [15].

It is crucial to remember that HPV infection is connected to almost every case of cervical cancer. However, some studies reveal the link of HPV infection to many distinct types of cancer in both sexes, including HNC, genital warts, and cancers of the vulvar, vaginal, anal, and penial systems. New anogenital warts occur at a rate of 137 per 100,000 males and 121 per 100,000 women per year, with a frequency of 0.15% to 0.18% in both sexes [16,17].

Insufficient reporting of data has the potential to obscure the actual prevalence and incidence rates of HPV in the Kingdom of Saudi Arabia (KSA). Therefore, the aim of this systematic review is to gather and evaluate the existing evidence pertaining to the correlation between HPV infection and cancer within the context of KSA. As per the estimation of the World Health Organization, a significant number of women aged 15 years and above in KSA, exceeding 6.5 million, are at a heightened risk of developing cervical cancer. In KSA, the HPV is responsible for around 55 fatalities per year in women due to cancers induced by the virus. The predominant HPV genotypes detected in the KSA comprise HPV-16, HPV-18, and HPV-45, and these genotypes cause 70% of cervical cancer cases in the country [18]. Most Saudi women with HPV-induced tumors delay seeking medical attention until the tumor has progressed to a point when drastic measures are needed, such chemotherapy, surgery, or radiation treatment. The problem is exacerbated by the reality that a majority of Saudi women are unaware of the seriousness of the illness, as shown by the fact that cytological screening of cervical samples is not frequent in the country [18]. Saudi women showed an unfavorable attitude about cervical cancer screening in relation to HPV. An investigation revealed that there is a dearth of knowledge on HPV, cervical cancer, and the need for screening [19]. There is a scarcity of national initiatives in KSA, which might lower rates of HPV prevalence and cancer compared to global rates [20]. The HPV vaccination coverage is unknown in KSA, and research indicates low HPV vaccination rates across various demographics. Studies show that only 2% of Saudi females received the vaccine in 2020, with a similar low uptake observed among both females and males in the Eastern Province and Riyadh with 4% and 8.7%, respectively.

HPV-related cancer rates and rates of exposure are strongly influenced by a community's racial and genetic make-up. There is a lack of comprehensive population-based epidemiological research on HPV-related malignancies in KSA. While individual epidemiological studies in KSA have looked at the connection between HPV and cancer, no comprehensive analysis has yet been conducted.

## Review

Methodology

This review followed the criteria for reporting systematic reviews and meta-analyses (Preferred Reporting Items for Systematic Reviews and Meta-Analyses [PRISMA]) [21].

Search strategy

For the purpose of searching the electronic databases, a comprehensive search strategy was adopted. The search terms and keywords were identified based on the research question. The databases used for the search of relevant studies included Embase, PubMed, Web of Science, Saudi Journal of Medicine and Medical Sciences, and Google Scholar till May 2023. The choice of these databases is based on their diverse and reputable coverage, with PubMed and Embase offering comprehensive biomedical literature, Web of Science providing a multidisciplinary perspective, the Saudi Journal of Medicine and Medical Sciences offering region-specific insights, and Google Scholar being inclusive of a wide range of scholarly publications, ensuring a thorough and comprehensive data extraction process. The search terms and combination of keywords included “prevalence OR incidence OR association OR relationship” AND “HPV OR human papilloma virus” AND “cervical OR ovarian OR head and neck OR oral OR colorectal” AND “cancer” AND “Saudi Arabia.” To discover pertinent literature, the bibliographies of the eligible studies were searched.

Eligibility and exclusion criteria

This systematic review included studies conducted on individuals residing in KSA, encompassing cross-sectional, case-control studies, and cohort studies reporting the prevalence or associations between HPV and various types of cancer. Additionally, the review considered studies demonstrating the prevalence, genotypes, and relationships between HPV and various kinds of cancers, with the inclusion criterion of studies published in the English language. Excluded from the analysis were research investigations in the form of reviews, conference abstracts, or case series, as well as those not written in English. The systematic review specifically incorporated case-control and cohort studies that fulfilled the specified eligibility criteria. The PECO criteria for this review consisted of the following: Population = Cancer patients, Exposure = HPV infection, Control = Patients without HPV, and Outcome = Presence of cancer.

Keywords

The combination of keywords that were used for the literature search included the following: Association OR relationship OR incidence OR prevalence of HPV OR Human papillomavirus AND Cancer OR Cervical cancer OR Ovarian cancer OR colorectal cancer OR head and neck cancer.

Study selection process

Two independent reviewers (Khalil M. Ismail and Tassnym H. Sinky) screened the chosen articles' titles and abstracts in accordance with the inclusion criteria. Then, the full-text papers of research that could be relevant were obtained and evaluated for eligibility. The selection of studies was discussed, and, if required, a third reviewer (Mashael S. Alfaifi) was consulted to settle any disagreements. Reviewer consistency was maintained through the establishment of specific criteria and regular meetings to address any discrepancies, thereby enhancing inter-rater reliability.

Data extraction

The following particulars were collected from the designated investigations, encompassing the authors, year of publication, research methodology, size of the sample, average age of the subjects, clinical specimens, type of cancer, HPV association, and outcomes. Any disputes between reviewers were resolved via discussion until agreement was reached.

Risk-of-bias assessment

The risk-of-bias assessment for the studies included in this analysis employed multiple tools. In observational research, including case-control and cohort studies, the Newcastle-Ottawa Scale (NOS) is frequently used to evaluate the quality and risk of bias. It assesses three areas: selection of research groups, group comparability, and determination of exposure/outcome [22]. For the assessment of cross-sectional studies, “Appraisal tool for Cross-Sectional Studies” (AXIS) was used [23].

Results

**Figure 1:**
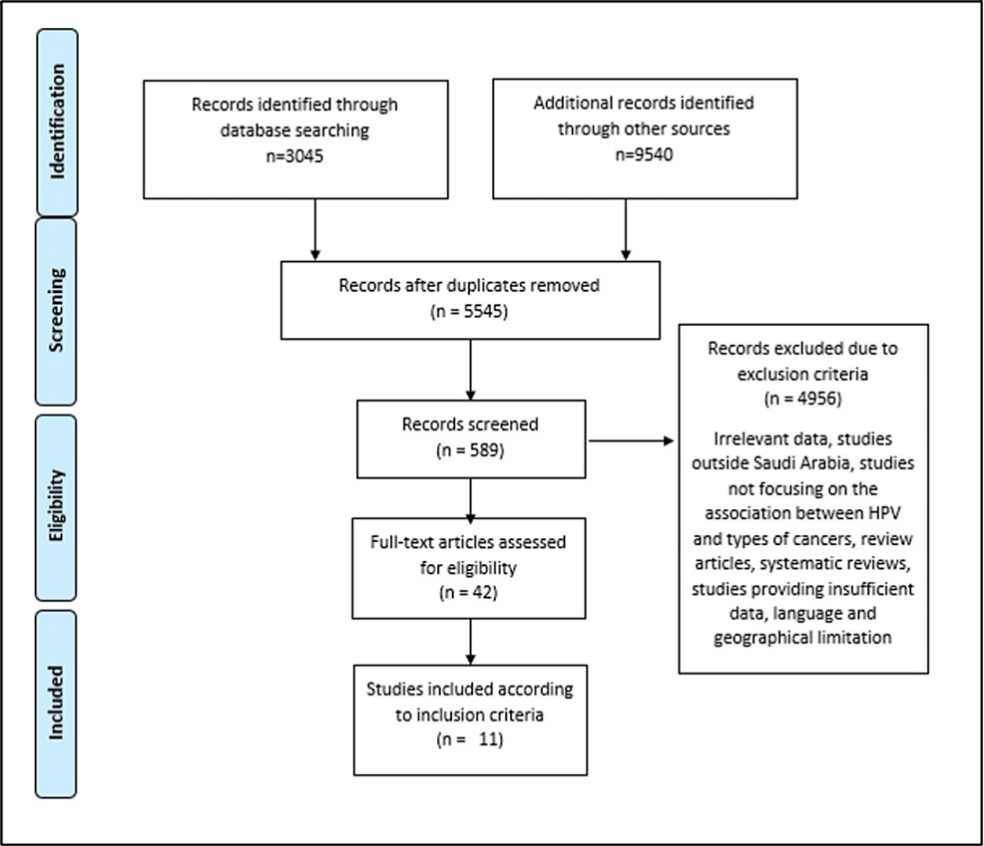
Flowchart for studies selection according to the selection criteria.

The search performed through the databases retrieved 12,585 articles. After removing the duplicates, 5,545 articles remained, which had their abstracts and titles screened. The full-text articles assessed were only 589 articles, and among them only 11 articles met the inclusion criteria. The exclusion of articles from this review was guided by a set of criteria, which encompassed a range of factors such as the presence of irrelevant data, studies conducted beyond the geographical boundaries of the KSA, studies that did not specifically explore the correlation between HPV and various types of cancers, review articles, systematic reviews, studies that presented inadequate data, and limitations pertaining to language and geography. Figure 1 represents the flowchart for study selection according to the selection criteria.

Risk-of-bias assessment

The risk of bias was assessed by using two tools: NOS and AXIS. The results are presented in Tables 1-3. The NOS provides up to nine points for a minimal risk of bias in three domains: 4 points for study group selection, 2 points for group comparability, and 3 points for exposure and outcome assessment for the case-control and cohort studies [24]. It has eight items spread over three domains, and the top score possible is 9. A study with a score of 7-9 has a high risk of bias, that with a score of 4-6 has a high quality risk, and that with a score of a 0-3 has an extremely high risk. In the AXIS tool, each question in the evaluation tool offers an option to record a "yes," "no," or "don't know" response for 20 questions [23]. All the studies included in this systematic review were of moderate-to-good quality.

**Table 1:** Quality assessment of case-control studies using the Newcastle-Ottawa Scale (1 for yes and 0 for no)

	Selection			Comparability	Exposure			
Studies	Accurate case definition	Representativeness of case	Control selection	Comparability of cases and controls	Ascertainment of exposure	Same method for ascertainment	Non-response rate	Total
Gazzaz et al. [25]	1	1	0	1	1	1	1	6
Almarzooqi et al. [26]	1	1	1	1	1	1	1	7
Alsbeih et al. [27]	1	1	1	1	1	1	1	7

**Table 2:** Quality assessment of cohort studies using the Newcastle-Ottawa Scale

	Selection				Comparability	Outcomes			
Studies	Representativeness of cohort	Selection of non-exposed cohort	Ascertainment of exposure	Outcome demonstration at the start of the study	Comparability of cohorts	assessment	Follow-up long enough	Adequacy of follow-up	Total
Alsbeih et al. [28]	1	1	1	1	1	1	1	1	8

**Table 3:** Quality assessment of cross-sectional or descriptive studies using the “Appraisal tool for Cross-Sectional Studies” (AXIS) tool (Y for yes, N for no, and ? for don’t know)

Studies	1	2	3	4	5	6	7	8	9	10	11	12	13	14	15	16	17	18	19	20
Alsbeih et al. [29]	Y	Y	Y	Y	Y	Y	Y	Y	Y	Y	Y	Y	Y	Y	Y	Y	Y	Y	Y	Y
Al-Shabanah et al. [30]	Y	Y	Y	Y	Y	Y	Y	Y	N	Y	N	Y	Y	Y	Y	Y	Y	Y	Y	Y
Gazzaz [31]	Y	Y	Y	Y	Y	Y	Y	Y	Y	Y	Y	Y	Y	Y	Y	Y	Y	Y	Y	Y
Al-Muammar et al. [32]	Y	Y	Y	Y	Y	?	Y	Y	Y	Y	Y	?	N	Y	Y	Y	Y	Y	Y	Y
Al-Badawi et al. [33]	Y	Y	Y	Y	Y	Y	Y	?	Y	Y	Y	Y	Y	Y	N	Y	Y	Y	Y	Y
Jaber et al. [34]	Y	Y	Y	Y	Y	Y	Y	?	Y	Y	Y	Y	Y	Y	Y	Y	Y	Y	Y	Y
Ahmed et al. [35]	Y	Y	Y	Y	Y	Y	Y	Y	Y	Y	Y	Y	Y	Y	Y	Y	Y	Y	Y	Y

Characteristics of studies

The studies incorporated in this comprehensive systematic review spanned from 2007 to 2023. The included studies involved 1,386 patients with various types of cancers. Among various cancer types, five studies reported cervical cancer, one study reported ovarian cancer, three studies reported HNC, one study reported colorectal cancer, and one study reported oral cavity cancer. The mean age range of participants included in the studies was 37-61 years. Among the included studies, three were case-control studies, one was a cohort study, and seven were cross-sectional or descriptive studies. Table 4 shows the characteristics of the included studies.

**Table 4:** The characteristics of the included studies HPV, human papillomavirus

Authors	Year	Type of study	Region	No. of patients	Clinical specimens	Mean age	Cancer type	Patients with HPV detection	Outcomes
Alsbeih et al. [29]	2011		Riyadh	100	Paraffin-embedded cervical biopsies	46	Cervical cancer	89	89% of cervical cancer is associated with HPV
Gazzaz et al. [25]	2016	Case-control study	Jeddah	132	Colonoscopic biopsies	53	Colonic polyps and colorectal cancer	4	Colonic colonization of HPV is rare
Al-Shabanah et al. [30]	2013	Original research	Riyadh	100	Formalin-fixed paraffin-embedded ovarian tissues	50	Ovarian cancer	42	HPV genotypes have a role in ovarian carcinogenesis
Alsbeih et al. [27]	2013	Research article	Riyadh	100		46	Cervical cancer	82	HPV prevalence is associated with cervical cancer
Alsbeih et al. [28]	2019		Riyadh	285	Pathologic materials	57	Head and neck cancer	10	Low prevalence of HPV infection in head and neck cancer
Gazzaz [31]	2007	Clinical trial	Jeddah	100	Cervical cytological samples	42	Cervical cancer	5	Patients who have been infected with HPV for many years are unlikely to develop cancer
Al-Muammar et al. [32]	2007		Riyadh	120	Pap smear specimens	37	Cervical intraepithelial neoplasia	13.30%	High HPV prevalence but low progression of neoplasia
Al-Badawi et al. [33]	2011		Riyadh	100	Clinical samples of cervical cancer	56	Cervical cancer	95.50%	Percentage of cervical cancer associated with HPV is less than that of the international figures
Jaber et al. [34]	2019		Dammam	45	Paraffin-embedded tumor blocks	61.2	Oral cavity squamous cell carcinomas	41	High-risk HPV is not involved in the etiology of cancer
Ahmed et al. [35]	2012	Retrospective study		150	Formalin-fixed paraffin-embedded tissues	54	Head and neck cancer	31	Prevalence of HPV is high among patients with head and neck cancer
Almarzooqi et al. [26]	2023	Case-control study	middle east	154	Histopathological slides samples	59	Head and neck squamous cell carcinoma	5	HPV plays a minor role in developing cancer

The study by Alsbeih et al. found that more than 90% of the cases were HPV-positive, indicating a widespread infection. HPV-16, HPV-31, HPV-45, and HPV-18 were the most genotypes of HPV often found. When compared to other HPV genotypes, HPV-16/18 was shown to be linked to an earlier onset of cervical cancer [29]. These results highlight the need for HPV vaccination and screening programs in KSA that emphasize on high-risk genotypes, especially HPV-16 and HPV-18, to prevent cervical cancer and its early start.

 Gazzaz et al. assessed the incidence of HPV colonization in the colon. The results showed that only 4% (0.8%) of the samples tested positive for the HPV gene. CRPs or colorectal cancer were not statistically significantly correlated with HPV colonization [25]. These results show that colonic HPV colonization is uncommon in the population under study and that other variables might have a more substantial role in the etiology of colonic polyps and colorectal cancer.

Al-Shabanah et al. investigated the prevalence and genotyping of HPV involved in ovarian cancer. Compared to the surrounding normal tissues, ovarian cancer was shown to have a much higher HPV prevalence (42% vs. 8%). Types 16, 18, and 45 of high-risk HPV were linked to more advanced tumor stages, whereas types 6 and 11 of low-risk HPV were discovered in normal tissues. Malignant tissues tended to have a high incidence of HPV-16. Most HPV-positive tumor tissues (61.1%) exhibited integration of HPV into the host genome [30]. These results emphasize the relevance of HPV vaccination in preventing ovarian cancer and point to a possible involvement of high-risk HPV genotypes in ovarian carcinogenesis.

Alsbeih et al. assessed the incorporation of HPV in cases of cervical cancer. This study involved a cohort of 100 individuals diagnosed with cervical cancer and 100 healthy controls, matched for age and sex. The prevalent HPV genotype observed in cervical cancer cases was HPV-16, with HPV sequences being detected in 82% of the cases. Cervical cancer was shown to be strongly related to XRCC1 G399A, whereas TP53 G72C showed only a weak connection in HPV-positive individuals. The presence of a greater number of polymorphisms in HPV-positive individuals than anticipated reveals an interplay between HPV and single nucleotide polymorphisms (SNPs), suggesting a combined effect on cancer susceptibility [27]. These results indicate that SNPs may function as useful indicators for cervical cancer susceptibility when HPV infection is taken into account.

A study by Alsbeih et al. evaluated the prevalence of HPV in HNC patients. There were 285 patients with head and neck squamous cell carcinoma (HNSCC) in their study. Oropharyngeal malignancies accounted for the vast majority of instances in which HPV-DNA was found (3.5%). Although 42% of patients showed overexpression of the HPV-associated p16INK4a (p16) protein, this was substantially correlated with better overall survival (OS). Significant variables influencing OS were age, smoking status, tumor stage, and therapy. The study found that individuals who tested positive for HPV/p16 exhibited a notably extended OS compared to those who tested double negative for HPV/p16 [28]. Nevertheless, the available estimates indicate that the incidence of HPV in patients with HNSCC in KSA remains lower than the global average.

Gazzaz et al. performed a study involving 100 patients with cervical cancer. The aim was to enhance the detection of cervical neoplasia, and the screening methods used were hybrid capture 2 (HC2) test and polymerase chain reaction (PCR). The data showed that compared to PCR, HC2 test found 94% negative instances, 5% high-risk HPV, and 1% low-risk HPV. Through a series of comparisons with the HC2 test, the Pap test's sensitivity, specificity, and accuracy were calculated. Those who had abnormal or negative Pap findings but positive HPV DNA were given a follow-up Pap test [31]. The study findings indicate that the detection of preexisting medical conditions could potentially be more dependable through the implementation of a combined screening approach involving cytology and HPV testing using HC2 and PCR. Patients may be certain that their cancer risk will be reduced in the years ahead if no HPV DNA is detected. The research suggests adding Pap tests to screening programs alongside HC2 and PCR, which would allow for longer intervals between screens for those who first test negative.

Al-Muammar et al. measured the prevalence of HPV-16 and HPV-18 infections in cervical tissues. The study found that the prevalence of HPV-16 was 13.3%, while the prevalence of HPV-18 was only 3.3%. Around 15% of HPV infections were found to include both HPV-16 and HPV-18. Pap smears revealed abnormalities in the cervical region in 10 people, of whom six people tested positive for HPV-16/18. Over the course of four years, 23 people who tested positive for HPV-16/18 were followed up, and seven of them had aberrant cytology at some point. Except for one instance that needed therapy and eventually returned to normal after three years, all seven cases resolved without intervention. The individuals did not develop CIN-III (cervical intraepithelial neoplasia grade III) [32].

Another study by Al-Badawi et al. ascertained the incidence and typology of HPV infection in cases of cervical cancer. According to the analysis conducted, it was discovered that the cervical cancer samples tested exhibited the presence of HPV genotypes in 95.5% of the cases. The study revealed that HPV-16 was detected in 63.4% of cases, while HPV-18 exhibited a prevalence of 11.1%. Additionally, HPV-45 was present in 4.5% of cases, HPV-33 in 3.3%, and a few other genotypes were found in 2.2% of cases [33].

Jaber et al. investigated potential association between HPV and oral cavity carcinoma. The study employed p16 immunohistochemistry and in situ hybridization for DNA detection to assess the prevalence of high-risk HPV strains, including types 16, 18, 31, and 33. The study involved the participation of 24 male individuals with a mean age of 59.3 and 21 female individuals with a mean age of 61.2. Out of the total 55 samples, 14 exhibited negative results for p16 immunostaining, while the remaining 41 samples showed positive results. None of the 45 cases examined exhibited DNA expression for any of the HPV subtypes investigated [34]. These findings indicate that high-risk HPV is probably not a major contributor to the emergence of oral cancer.

Ahmed et al. evaluated the presence of HPV in cases of HNC. A retrospective analysis was conducted on a sample of 150 individuals diagnosed with cancer. The use of the p16INK4A protein expression was employed to determine the HPV status. Among the 150 HNSCC samples analyzed, 27 of them, accounting for 20.7%, exhibited positivity for HPV [35]. The results suggest that a considerable proportion of cases of HNC are linked to the presence of HPV.

Almarzooqi et al. conducted an investigation into the prevalence of high-risk HPV in cases of HNC. The results indicated that a significant proportion of the 8.6% of cases that exhibited high-risk HPV positivity comprised oropharyngeal cancers [26]. Based on the results of this study, it can be inferred that the incidence of HNC in the region under investigation was primarily attributed to cigarette and alcohol consumption, rather than high-risk HPV infection.

Overall, the results show that there is a strong association between HPV and cervical cancer and suggest logical connections with squamous cell carcinomas of the colon, ovaries, and head and neck. The data indicated above highlight the need for further investigation into the role of HPV in the initiation and progression of these cancers. Additional research is required to improve our understanding of HPV's involvement in these tumors.

Discussion

The HPV prevalence, genotypes, and clinical consequences in different types of cancers were investigated in the included studies performed in KSA. The findings consistently demonstrated a significant presence of HPV in cervical cancer, underscoring the imperative for HPV screening and immunization initiatives aimed at preventing cervical cancer. This research is poised to pave the way for future studies, bringing about a substantial impact on public health and clinical practices within the same field in KSA. Although HPV is the sole significant risk factor for cervical cancer, several experts contend that the immune system can typically eliminate the infection without any complications as viral DNA integration is infrequent.

After integration, viral DNA may rapidly change infected cells into neoplastic ones, although it likely takes more than HPV DNA being present in the cell to produce cancer [36]. Two key carcinogenic proteins produced by HPV, E6 and E7, affect the regulation of apoptosis and cell cycle control. The E2 protein's function is altered due to the inclusion of viral DNA. Dysregulated production of oncoproteins E6 and E7 results from the loss of the E2 protein, which is known to block their transcription. These proteins work together to prevent cell death, allowing the cells to divide continuously and produce cells with the same immortalized phenotype [37,38]. Combating HPV infection and preventing cervical carcinogenesis relies heavily on the immune response. However, by expressing the E5 oncogene, HPV is able to enhance immune evasion by modulating a number of immunological processes, such as presenting antigens and inflammatory pathways [39]. Understanding how HPV interacts with the immune system and uses the E5 oncogene's evasion mechanisms offers insights for developing preventive and therapeutic strategies, including immunomodulation and antiviral approaches. Tailoring vaccination programs and early detection methods based on these insights can enhance efforts to combat HPV and prevent cervical cancer. A systematic review on the correlation between HPV-16/18-positive lesions and prognosis in cervical cancer revealed that there was no such correlation. This worked opposite to what was known about HPV-16/18-positive lesions in cervical cancer development, which is that they tend to be aggressive [40].

Additional research is necessary to explore the correlation between HPV infection and colorectal cancer as well as colon polyps, given the identification of the virus in these conditions. While the connection between HPV and colorectal cancer has been debated, there is strong evidence linking HPV to anal carcinoma, a rare tumor of the anus [41,42]. This finding was consistent with the findings of a comprehensive study that established a causal link between HPV and colon cancer in both sexes, without distinguishing between HPV types 16 and 18, and with equivalent values [43].

Specific HPV genotypes were found in ovarian cancer patients, and there was evidence of viral integration into the cancer cells, pointing to an oncogenic function for HPV in this situation. Ovarian carcinomas are known to have specific genetic changes. Ovarian cancer has been associated with a variety of risk factors, including personal or family history of the disease, advanced age, high ovulation rates, endocrine variables, endometriosis, pelvic inflammation, and a high-fat diet. HPV infection is not the only factor in the environment that has received attention recently [44-46].

According to a meta-analysis, the prevalence of HPV detection in ovarian cancer varies greatly across studies, although this variation is not evident in malignancies that are highly related to HPV, such as cervical cancer. However, there was a large disparity in occurrence across different regions [47]. The regional factors of HPV-related cancer development are further highlighted by the fact that the occurrence of HPV in HNSCC was much lower in this area than in other nations. Regional variations in HPV-related cancer rates can stem from cultural attitudes toward sexual health, socioeconomic factors affecting access to preventive healthcare, and differences in the prevalence of high-risk HPV strains. Understanding these variations is crucial for implementing targeted interventions. The likelihood of acquiring HPV-positive oropharyngeal malignancies is higher among those who have previously been exposed to the virus, while the risk of mouth cancer is lower after being diagnosed by seropositivity of HPV-16 [48].

Oral squamous cell carcinomas in Saudi patients do not contain HPV, suggesting a unique etiology for this disease. Several studies have indicated that while HPV DNA is present in a high proportion of mouth cancer cases, this illness is still considered to be a distinct clinical entity with many unanswered problems. It is not known if HPV-driven oral cavity cancers are HPV-positive since the presence of HPV-DNA does not confirm the occurrence of a biologically active HPV [49]. In summary, the aforementioned findings underscore the necessity for comprehensive HPV screening, vaccination, and further investigation to enhance our understanding of the correlation between HPV and various forms of cancer in KSA.

To the best of our knowledge, this represents the first investigation conducted in the KSA, which was performed to find out the prevalence of HPV across various malignancies. This systematic review has a number of limitations. First of all, the research made it difficult to make conclusions on the prevalence of cancer since neither a control group nor a large enough sample size was used. Secondly, HPV genotypes were not disclosed by histological type in several studies. This finding emphasizes the need for more research that differentiates cancer according to its histologic subtype. The prevalence of HPV in cancer in KSA could not be conclusively determined due to inconsistencies in the age range of the studies included in the analysis. The majority of the studies incorporated in the analysis solely presented the average age of the investigated cohort.

## Conclusions

The findings indicate a significant correlation between HPV infection and the onset of cervical cancer, as evidenced by the high incidence of HPV detection among patients diagnosed with invasive cervical cancer in KSA. This finding underscores the urgent need for targeted public health interventions and clinical practices. Enhanced public health awareness campaigns should emphasize HPV vaccination and regular cervical cancer screenings to mitigate the disease's impact. Implementing comprehensive screening programs, integrating HPV vaccination into national immunization schedules, and training healthcare providers on counseling and guidelines are crucial steps. Additionally, ongoing research and surveillance efforts are necessary to monitor HPV prevalence, evaluate intervention effectiveness, and inform evidence-based policies. By addressing these practical implications, KSA can effectively reduce the burden of cervical cancer associated with HPV infection, ultimately improving population health outcomes. The presence of polyps in the colon and colorectal cancer has been linked to HPV infection. This finding presents additional support for the potential association between HPV and the onset of colorectal cancer, emphasizing the need for deeper research into viral contributions to gastrointestinal malignancies. This understanding could pave the way for innovative preventive measures such as targeted vaccinations and specialized screening protocols for HPV-infected individuals at risk of colorectal cancer. A study conducted on patients from the KSA has discovered evidence that establishes a connection between ovarian cancer and HPV integration and genotyping. The potential association between HPV and ovarian cancer has been identified, which may contribute to a better understanding of the etiology of this malignancy. Furthermore, research has demonstrated a correlation between HPV and malignancies in the head and neck region. The present investigation provides valuable insights into the intricate relationship between HPV and cancer. The precise contribution of HPV in various cancers such as those affecting the colon, ovaries, and head and neck remains uncertain. Further investigation is warranted regarding the various types of cancer and their corresponding demographics in order to enhance comprehension of the mechanisms underlying HPV-related carcinogenesis. Further investigation, including population-based studies and molecular research, is needed to elucidate the precise contribution of HPV to cancers affecting the colon, ovaries, and head and neck. Population-based studies would provide valuable insights into the prevalence and incidence rates of HPV-related cancers across different demographics, helping in identifying high-risk populations and inform targeted prevention strategies. Molecular studies could elucidate the mechanisms underlying HPV-related carcinogenesis in these specific cancer types, potentially leading to the development of targeted therapies and personalized treatment approaches.
